# A Pilot Study on Dietary Approaches in Multiethnicity: Two Methods Compared

**DOI:** 10.1155/2012/985136

**Published:** 2012-03-15

**Authors:** Romina Valentini, Maria Grazia Dalfrà, Michela Masin, Antonella Barison, Marcon Marialisa, Eva Pegoraro, Annunziata Lapolla

**Affiliations:** Department of Medical and Surgical Sciences, U.O. of Metabolic Diseases and Diabetology, University of Padua, Via Giustiniani 2, 35143 Padua, Italy

## Abstract

*Background*. Medical nutritional therapy is the most important method for normalizing glucose levels in pregnancy. In this setting, there is a new problem to consider relating to migrants, their personal food preferences, and ethnic, cultural, and religious aspects of their diet. We compared maternal and fetal outcomes between two multiethnic groups of pregnant women, one adopting a food plan that included dishes typical of the foreign women's original countries (the “ethnic meal plan” group), while the other group adopted a standard meal plan. *Findings*. To develop the meal plan, each dish chosen by the women was broken down into its principal ingredients. The quantity of each food was given in tablespoons, teaspoons, slices, and cups, and there were photographs of the complete dish. The group treated with the ethnic meal plan achieved a better metabolic control at the end of the pregnancy and a lower weight gain (though the difference was not statistically significant). As for fetal outcome, the group on the ethnic meal plan had babies with a lower birth weight and there were no cases of macrosomia or LGA babies. *Conclusions*. This preliminary study indicates the positive effect of an ethnic approach to diet on the outcome of pregnancy.

## 1. Introduction

Gestational diabetes (GDM), that is, carbohydrate intolerance first recognized and/or diagnosed during pregnancy [1], is the most common metabolic complication of gestation, with a prevalence estimated at around 14% [2, 3]. Obesity, family history of diabetes, and belonging to certain ethnic groups increase the risk of GDM [4–11]. If the disorder is not properly monitored and treated, it can cause severe complications in both mother (including preeclampsia, cesarean delivery, glucose intolerance, or type 2 diabetes after delivery) and child (macrosomia, hypoglycemia, hyperbilirubinemia, adolescent obesity, glucose intolerance, and diabetes) [2, 4, 12, 13].

Immigration rates have recently increased in Italy, and immigrants now account for about 4% of the resident population; 48% of the immigrant population consists of women, and 65% of them are of reproductive age [14]. Some studies have demonstrated a tendency for adverse outcomes of pregnancy among immigrant women from countries with high rates of diabetes [5–8].

The first step in treating GDM is nutrition therapy [1, 15]. Immigrants differ in their cultural background and eating habits, so we aimed to assess such women's compliance with dietary restrictions and the possible benefit, in terms of maternal and fetal outcome, of adopting a nonstandard, ethnic-based approach to their diet.

## 2. Materials and Methods

For this pilot study, twenty pregnant immigrant women with GDM followed up at the Metabolic Disease and Diabetology Unit of Padova University between January and June 2008 were enrolled. The study protocol complied with the Helsinki Declaration and was approved by the local Ethics Committee, and written informed consent was obtained from all participants.

Screening for GDM was done with a glucose challenge test (GCT) between weeks 24 and 28 of gestation, and the diagnosis was confirmed with a 100 g oral glucose tolerance test (OGTT) as recommended by the 4th International Workshop Conference on GDM [1].

The women enrolled were randomly assigned to two groups: one adopted a standard meal plan (SMP) prepared according to the ADA guidelines [15], the other an ethnic meal plan (EMP) (Table 1).

All the women were monitored to achieve a good metabolic control, that is, fasting plasma glucose (FPG) <5.3 mmol/L and 1 h postprandial plasma glucose (1 h PPPG) <7.2 mmol/L, and nurses taught them to monitor their own blood glucose levels [15]. The pregnant women on diet treatment performed 2 measurements per day, measuring fasting and 1 h postprandial glucose on alternate meals over the course of a week. The women on insulin therapy performed self-glucose monitoring four times a day (fasting and 1 h after breakfast, lunch, and dinner). They saw a specialist every two weeks. Insulin treatment was started when FPG and/or 1-hour PPPG exceeded the above level in more than one measurement [15]. All GDM women were followed up for metabolic and obstetric purposes until delivery.

For maternal characteristics and outcome, we considered age, prepregnancy body mass index (BMI, kg/m^2^), time of GDM diagnosis, HbA1c (when GDM was diagnosed and at delivery), percentage of patients on insulin, weight gain, timing and mode of delivery, and hypertensive disorders.

For fetal outcome, we considered birth weight, infants large or small for gestational age (LGA, SGA), and fetal composite outcome (hypoglycemia, neonatal asphyxia, respiratory distress syndrome, and hyperbilirubinemia, hypocalcemia) and fetal malformations. Babies were LGA if their birth weight was above the 90th percentile and SGA if it was below the 10th percentile according to population-specific standard growth tables [16]. Macrosomia was diagnosed for a fetus weighing more than 4000 g.

A dietary assessment was conducted to determine whether a woman's intake of essential nutrients was adequate and whether she was eating excessively and to identify foods she avoided, as well as food intolerances or allergies. A meal plan was then developed, and patient and dietician prepared a sample menu. Food models, using measures in cups, glasses, and bowls, proved helpful props when teaching appropriate serving sizes.

The two groups received different meal plans: group 1 adopted the SMP for GDM according to the ADA guidelines [15]; group 2 adopted the EMP, which included typical foods of the women's home countries, identified using a photographic atlas (Dietmeter and Photographic Atlas, Scotti Bassani) [17–19].

**Figure 1:**
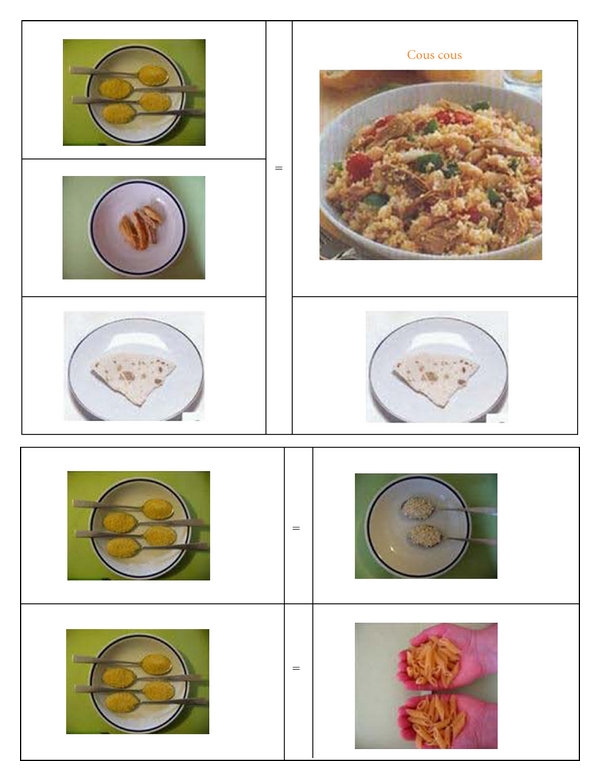
Example of ethnic meal plan.

The EMP included foods commonly consumed by patients according to their ethnicity. Dishes were broken down into the various ingredients, shown raw and cooked. Due to difficulties in using kitchen scales, measures such as cups, or spoonfuls handfuls or pinches, were preferred (Figure 1). Furthermore, the food pyramids of the specific country of origin were used.

The two meal plans had the same nutrient composition (SMP: CHO 53%, L 28%, P 18% fiber 26 g; EMP: CHO 55%, L 28%, P 17%, fiber 21 g), and energy intake was from 1800 to 2200 Kcal, depending on prepregnancy BMI.

Adherence to the diet was measured using a 24-hour food intake recall method and scored as 0 for an intake more than 20% higher than prescribed, 1 if the intake was 10–20% higher; 2 if it was consistent with the plan or up to 10% lower. The intake was calculated in individual tables, based on the INRAN nutritional tables, 2000 version [20].

Plasma glucose was evaluated using the glucose-oxidase method [21]. HbA1c was measured using standard HPLC; the normal range assumed for healthy pregnant women was 4.0–5.5% (20–37 mmol/mol) [22].

### 2.1. Statistical Evaluation

Data are given as means ± standard deviations and were compared using Student's *t*-test for unpaired data or for paired data when comparisons were drawn at different times in the same sample. The groups were compared for categorical data or frequency of an event using the *χ* ^2^ test with Yates' correction. A *P* value of less than 0.05 was considered significant. The data were processed using the SPSS18 for Windows program.

## 3. Results

### 3.1. Clinical and Metabolic Features

The ethnic distribution in EMP group was Chinese 1; Filipino 1; Moroccan 1; Nigerian 3; Romanian 4; Bangladeshi 1. In SMP group, was Chinese 1; Moroccan 1; Nigerian 1; Romanian 4; Sudanese 1, Bangladeshi 1, Hungarian 1, with no difference between the two meal plan groups.

**Figure 2:**
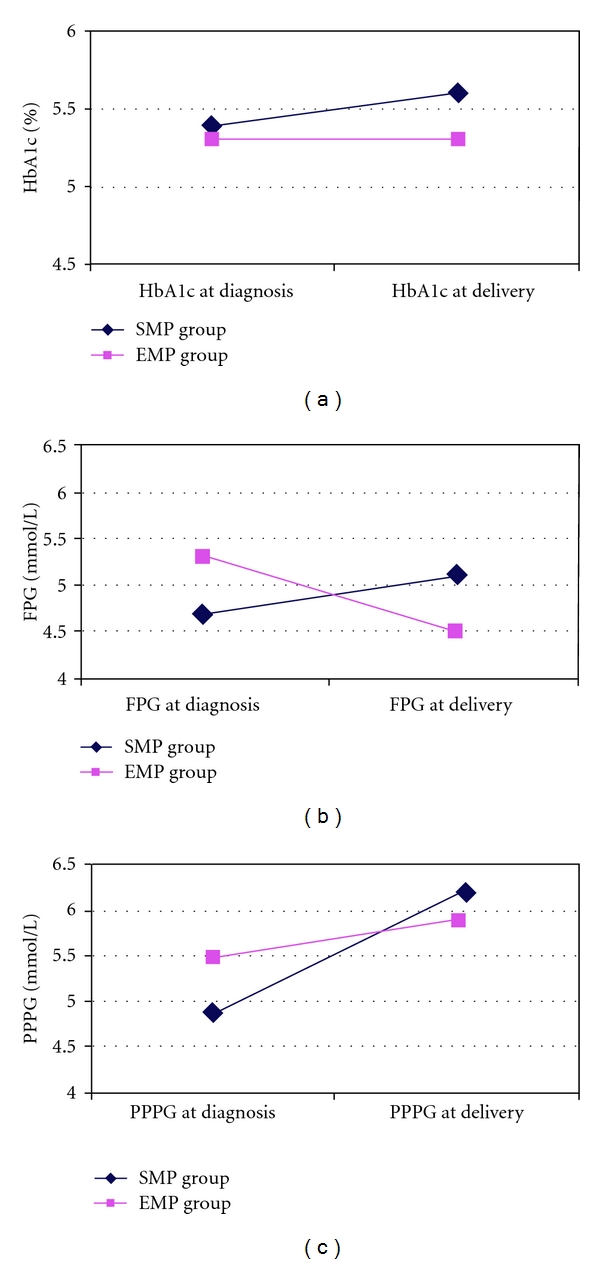
Metabolic parameters ((a) HbA1c; (b) FPG; (c) PPPG) evaluated in the subjects under study, AMP group: standard meal plan; EMP group: ethic meal plan; FPG: fasting plasma glucose; PPPG: 1-hour postprandial plasma glucose.

Table 2 shows the women's clinical and metabolic characteristics, and maternal and fetal outcomes. Mean age, prepregnancy BMI, and time of GDM diagnosis were comparable for the two groups. The EMP group had better FPG, 1hPPPG, and HbA1c values than the SMP group. Weight gain was lower, though not significantly, in the EMP group (Figure 2).

### 3.2. Pregnancy Outcome

No differences in maternal outcome emerged between the two groups. The newborns' birth weight was slightly higher in the SMP group, which also included more LGA (3 versus 0, *P* < 0.001) and macrosomic babies (2 versus 0) than the EMP group. There were no cases of SGA babies. No fetal complications or congenital malformations were seen in either group (Table 2).

### 3.3. Adherence to Dietary Recommendations

Adherence to the meal plans was better in the EMP group, with 7 women scoring 1 or 2 (good adherence), as opposed to 2 women in the SMP group. This difference was statistically significant (*χ* ^2^ = 0.025). 

## 4. Discussion

Immigration flows have increased in Italy, meaning rising numbers of pregnant immigrant women [14]. Italian data [14] on pregnancy indicate a relatively poor outcome of such pregnancies due to difficulties in accessing care and following medical recommendations for cultural and social reasons. Some immigrant populations are also at higher risk of type 2 diabetes and gestational diabetes [2, 8, 10]. 

Better standards of living in host countries often have negative effects on the health of immigrant populations, partly because foreign citizens acquire eating habits and lifestyles unsuited to their genetic profile. Regarding GDM, the problem is exacerbated by a genetic predisposition of African, Afro-American, and Hispanic races to develop diabetes [4, 8, 9].

Nutritional therapy for immigrant women should take the social, cultural, and religious value attached to food by the various ethnic groups into account.

In our study, the main problems the women had with their prescribed diet were related to difficulties in changing their eating habits; problems with managing a meal plan and weighing the foods; doubts concerning which foods to choose; difficulties in achieving the right nutritional balance.

On the other hand, many studies have confirmed the important influence of dietary treatment on the outcome of GDM [12].

The real difference between the two meal plans we used lies in how the diet and examples of typical dishes are presented [23]. The meal plan containing new elements adapted to different ethnic and cultural needs, with photos and practical domestic units of measure to illustrate the quantities of each food, plus lists of alternative foods and photos showing the foods raw and cooked proved useful and effective for our immigrant women with GDM, since adherence was better among the patients adopting the EMP.

Better adherence coincided with better glycemic control, a more normal weight gain [24], and a better pregnancy outcome, that is, the birth weights were lower, and there were no LGA or SGA babies in the EMP group.

The weakness of our study lies in the small numbers of patients considered but, to the best of our knowledge, this is the first study on the feasibility and efficacy of customized dietary treatment for immigrant GDM patients.

## 5. Conclusion

This pilot study indicates the positive effect of an ethnic approach to diet on the outcome of pregnancy. The new methods introduced in our study could be considered a valid approach to the nutritional management of immigrant pregnant women with gestational diabetes mellitus. It points out that, when prescribing a diet for immigrant women, their different traditional eating habits should be borne in mind. A study with an adequate number of women chosen in accordance with the power calculation is necessary and useful to confirm these preliminary data.

## Figures and Tables

**Table 1:** Example of two diets on 1800 kilocalories.

	Standard meal plan		Ethnic meal plan	
	Meal	Porzion size	Meal	Porzion size
Breakfast	Milk, skimmed	1 cup	Yogurt, skimmed	1
	White bread	1 slice	Crackers	4 slices

Snack	Apple	1	Mango	1

Lunch	Pasta	1 cup	Cous cous	1 cup
	Vegetables	1/2 cup	Vegetables	1/2 cup
	Meat	100 g	Poultry	3/4
			Bread without yeast	1 slices
	Olive oil	2 T	Sunflower oil	2 T

Snack	Yogurt, skim	1	Banana	1
	White bread	1 slice	Yogurt, skim	1

Dinner	Soup with:		Soup with:	
	Pasta	1/2 cup	Beans	1/2 cup
	Parmesan	1 T	Potatoes	One small
			Lentils	1/2 cup
	White bread	2 slice	Plantain	one
	Vegetables	1/2 cup	Vegetables	1/2 cup
	Mozzarella	100 g		
	Olive oil	2 T	Olive oil	2 T

Snack at bedtime	Milk, skimmed	1 cup	Yogurt, skimmed	1
	White bread	1 slice	Crackers	2 slices

T: tablespoon = 10 g; 1 cup liquid = 200 mL; 1 cup solid = 80 g; one slice = 30 g; Plantain = 100 g; 1 Fruit = 200 g.

**Table 2:** Clinical and metabolic characteristics and pregnancy outcome of the subjects under study.

	SMP group	EMP group	*P*
	*n* = 10	*n* = 10	
Age (yrs)	30.2 ± 4.7	28.9 ± 3.3	0,622
Prepregnancy BMI (kg/m^2^)	24.1 ± 4.7	25.7 ± 3.6	0,784
Time of GDM diagnosis (gw)	27.1 ± 5.9	21.3 ± 6.8	0,316
Weight gain during pregnancy (kg)	14.3 ± 6.9	12.1 ± 4.3	0,869
Insulin therapy (*n*)	1	2	0,509
Gestational hypertension (*n*)	1	0	0,330
Delivery (gw)	38.4 ± 1.1	38.0 ± 0.5	0,409
Cesarean section (*n*.)	5	6	1,000
Birth weight (g)	3434 ± 649	3064 ± 626	0,164
LGA babies (*n*)	3	0	0,001
SGA babies (*n*)	1	0	0,330
Macrosomia (*n*)	2	0	0,186
Fetal composite outcome (*n*)	0	0	—
Fetal malformations (*n*)	0	0	—

SMP: standard meal plan, EMP: ethnic meal plan, BMI: body mass index, LGA: large for gestational age, SGA: small for gestational age, gw: weeks of gestation.
